# Porcine Interferon Stimulated Gene 12a Restricts Porcine Reproductive and Respiratory Syndrome Virus Replication in MARC-145 Cells

**DOI:** 10.3390/ijms18081613

**Published:** 2017-07-25

**Authors:** Likai Ji, Xiang Zhou, Wan Liang, Jianjian Liu, Bang Liu

**Affiliations:** 1 Key Lab of Agricultural Animal Genetics, Breeding, and Reproduction of Ministry of Education & Key Lab of Swine Genetics and Breeding of Ministry of Agriculture, College of Animal Science and Technology, Huazhong Agricultural University, Wuhan 430070, China; jilikai01@126.com (L.J.); zhouxiang1986@gmail.com (X.Z.); wanleung828@webmail.hzau.edu.cn (W.L.); liujianjian@webmail.hzau.edu.cn (J.L.); 2 The Cooperative Innovation Center for Sustainable Pig Production, Wuhan 430070, China

**Keywords:** PRRSV, ISG12A, mitochondria, cell cycle, MARC-145 cells

## Abstract

Porcine reproductive and respiratory syndrome virus (PRRSV) causes severe losses in the global pig industry. In the present study, we investigated the molecular characterization of porcine interferon stimulated gene 12a (*ISG12A*) and confirmed its anti-PRRSV ability for the first time. We found that porcine ISG12A was localized in mitochondria and significantly decreased the number of cells in G2/S phase. Porcine *ISG12A* mRNA was up-regulated in cells/tissues of Tongcheng (TC) pigs and Large White (LW) pigs after PRRSV challenge. More importantly, the ectopic overexpression of *ISG12A* could significantly suppress PRRSV replication at 24, 36 and 48 h post challenge (hpc), which was confirmed by detecting PRRSV *ORF7* mRNA with quantitative reverse transcription polymerase chain reaction (qRT-PCR) and PRRSV N protein with indirect immunofluorescence assay (IFA) in MARC-145 cells. Meanwhile, knockdown of endogenic *ISG12A* could obviously facilitate PRRSV replication in MARC-145 cells at 36 hpc. The results will lead to a better understanding of the interaction between host immune system and PRRSV, which may help us develop novel therapeutic tools to control PRRSV.

## 1. Introduction

Porcine reproductive and respiratory syndrome (PRRS) is one of most economically devastating viral diseases in the global pig industry [1,2,3]. PRRS causes reproductive failure of late-gestation sows and respiratory diseases in young pigs [4]. The causative pathogen, porcine reproductive and respiratory syndrome virus (PRRSV), is a single-stranded positive-sense RNA virus. PRRSV is a member of small enveloped viruses and belongs to the order Nidovirales, Arteriviridae [5]. All PRRSV strains could be divided into two main genetic groups based on their remarkable genetic variation: PRRSV-1 (European) and PRRSV-2 (North America) [6]. The strains isolated in China belong to PRRSV-2 [7].

Interferons (IFNs) are an important family of cytokines secreted by host cells in response to different pathogens. There are three types of IFNs including IFN-I (IFN-α, IFN-β and several subtypes), IFN-II (IFN-γ) and IFN-III (IFN-λ1, IFN-λ2 and IFN-λ3). IFNs have potent pleiotropic biological activities, such as antiviral activity and immunomodulation [8]. Interferon-stimulated genes (ISGs) induced by IFNs play important roles in IFN signaling [9]. The protein products of ISGs have been demonstrated to restrict virus replication, such as RNaseL/2′-5′ oligoadenylate synthase (2′-5′OAS), double-stranded RNA protein kinase resource (PKR), Mx protein and nitric oxide synthase (NOS) [10,11,12]. Recently, Zhou and colleagues (2010) found that many ISGs were significantly induced by PRRSV infection, and some ISGs could significantly suppress PRRSV infection in MARC-145 cells, such as interferon stimulated gene 15 (*ISG15*), interferon induced protein with tetratricopeptide repeats 3 (*IFIT3*) and 2′-5′-oligoadenylate synthetase 1 (*OAS1*) [13,14,15].

Interferon-stimulated gene 12a (*ISG12A*) is a recently discovered as an antiviral ISG. Human *ISG12A* was initially cloned from human breast carcinoma cells (MCF-7) [16]. ISG12A protein encodes a small hydrophobic protein, which contains a conserved ~80 amino acid (aa) motif (the ISG12 motif) [17]. The promoter region of the *ISG12A* gene contains an IFN-stimulated response element (ISRE), interferon regulatory factor 1/2 (IRF1/IRF2) and signal transducer and activator of transcription (STAT) binding sites [18]. *ISG12A* can be induced by IFN-α, IFN-γ and polyinosinic-polycytidylic acid (poly(I:C)) [19]. ISG12A was identified as a pro-apoptotic protein involved in the caspase-dependent pathway [20]. As a member of the ISGs, *ISG12A* can dramatically restrict different viral infections, such as hepatitis C virus (HCV), vesicular stomatitis virus (VSV) and Newcastle disease virus (NDV) [21,22,23]. Whether porcine *ISG12A* is involved in host resistance to PRRSV infection is of great importance. In the present study, we analyzed its sequence characterization, subcellular localization, expression, function and anti-PRRSV ability. The results acquired from this study will contribute to further understanding of the interaction between the host and PRRSV, which may help us develop novel therapeutic tools to control PRRSV.

## 2. Results

### 2.1. Porcine ISG12A Sequence and Phylogenetic Analysis

**Figure 1:**
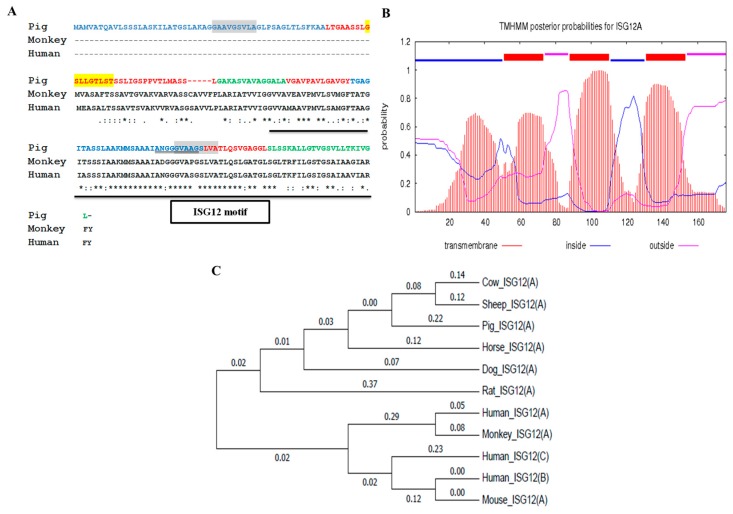
Sequence and phylogenetic analysis. (**A**) Interferon-stimulated gene 12a (*ISG12A*) homologues contain a conserved interferon-stimulated gene 12 (ISG12) motif (amino acid with underline), but porcine ISG12A has a unique amino acid sequence in its N terminal. Porcine ISG12A has four glycine zipper motifs (two GXXXGXXXA motifs with gray base color, one GXXXGXXXT motif with yellow base color and one AXXXGXXXG motif with double underline); “-”: without amino acid; “.”: weak similar amino acid; “:”: Strong similar amino acid; “*”: identical amino acid; (**B**) transmembrane helices of porcine ISG12A were predicted, probability >0.6. The transmembrane helices sequence was marked by red (Figure 1A); The inside sequence was marked by blue (Figure 1A); The outside sequence was marked by pink (Figure 1A); (**C**) phylogenetic tree showed the relationship between porcine *ISG12A* and homologues of nine other species.

The result of the multiple sequence alignment showed that porcine ISG12A protein contains a conserved ISG12 motif (95–169 aa), which is consistent with homologues of monkey and human (Figure 1A). Bioinformatics analysis with the TMHMM program (http://www.cbs.dtu.dk/services/TMHMM-2.0/) indicated that porcine ISG12A protein contains four glycine zipper motifs (two GXXXGXXXA motifs, one GXXXGXXXT motif and one AXXXGXXXG motif) and three putative transmembrane helices (Figure 1A,B). Phylogenetic tree according to DNA sequences showed that porcine *ISG12A* was closer to homologues of cow and sheep (Figure 1C).

### 2.2. Porcine ISG12A Is Located in Mitochondria and Regulate Cell Cycle

**Figure 2:**
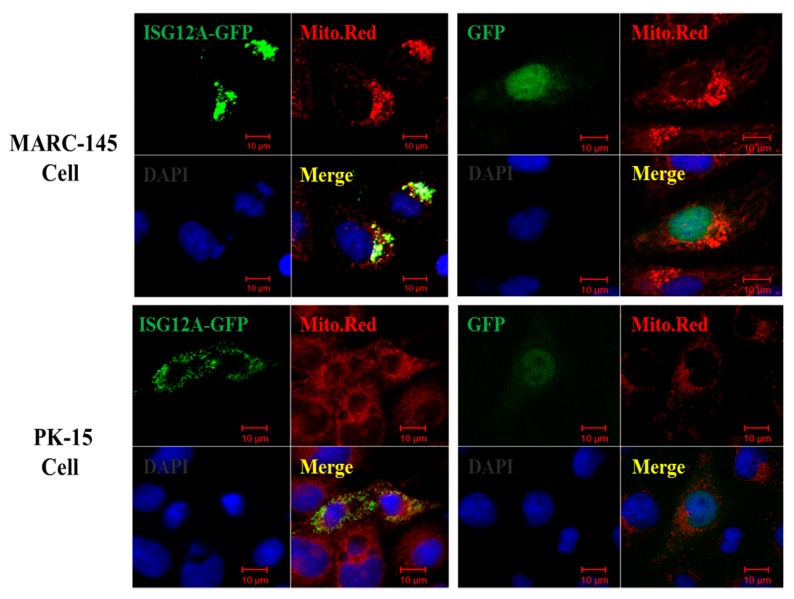
Subcellular localization of porcine ISG12A. MARC-145 or PK-15 cells were transfected with pEGFP-N1-ISG12A (ISG12A-GFP) or pEGFP-N1 (GFP, green) plasmid, and stained with MitoTracker^®^ Red for mitochondria (Mito. Red) and 4′,6-diamidino-2-phenylindole (DAPI, blue) for nucleus, Fluorescent images were acquired with a confocal laser scanning microscope (scar bar: 10 µm).

To investigate the subcellular localization of porcine ISG12A protein, the full-length coding sequence (CDS) of porcine *ISG12A* was cloned and was used to construct recombinant eukaryotic expression vector. Compared with control (green fluorescent protein, GFP), the results clearly showed that porcine ISG12A protein (ISG12A-GFP) was located in the mitochondria of both MARC-15 cells and PK-15 cells (Figure 2).

**Figure 3:**
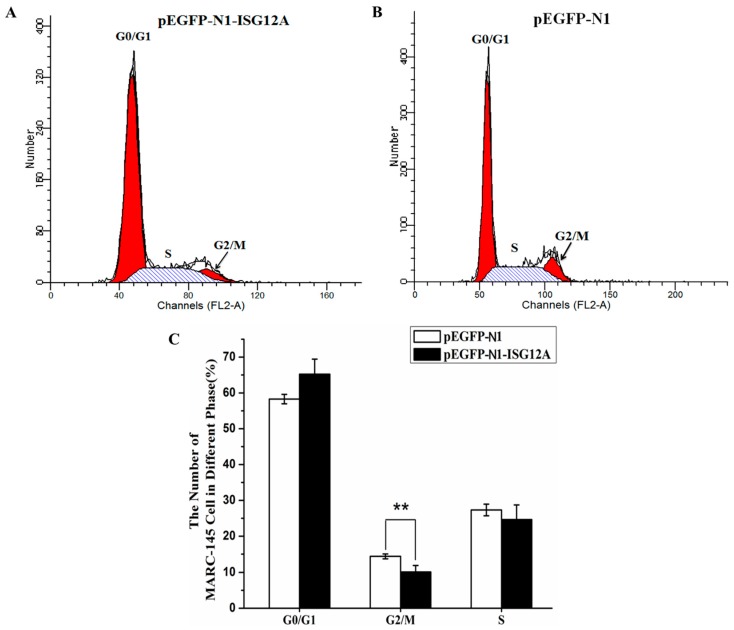
Porcine ISG12A influenced cell cycle. (**A**) DNA histogram measured by flow cytometry of the cells transfected with expression vector; (**B**) DNA histogram measured by flow cytometry of the cells transfected with control vector; (**C**) The statistical results of flow cytometry. G0/G1: first gap period; G2/M: second gap period; S: DNA synthesis and chromosome replication. Experiments were repeated three times. Student’s *t*-test: ** *p* < 0.01.

Mitochondria, as the energy provider in animal cell, can determine cell fate and is related to apoptosis in pathological conditions [24]. Flow cytometry-based cell cycle measurement showed that ectopic expression of porcine ISG12A could significantly reduce the number of MARC-145 cells in G2/S phase (Figure 3A–C).

### 2.3. Increased Expression of Porcine ISG12A after PRRSV Challenge In Vivo

**Figure 4:**
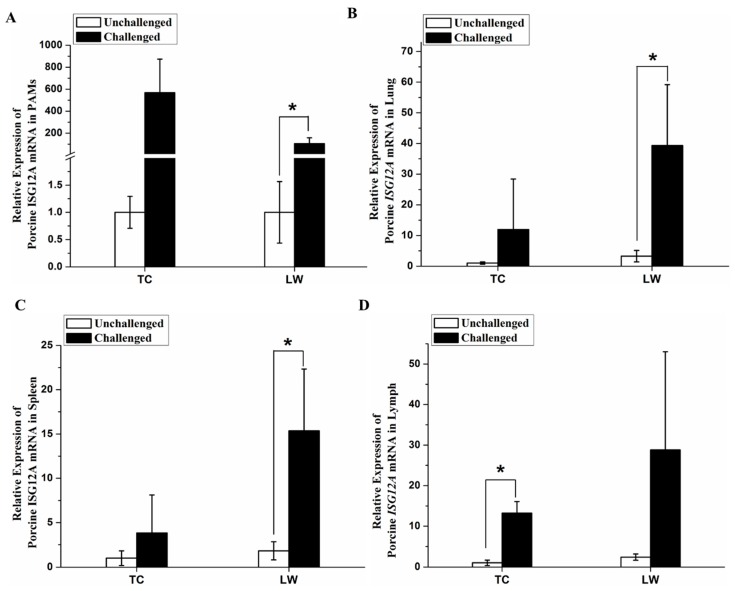
Porcine *ISG12A* mRNA expression was in different cells/tissues after PRRSV challenge. (**A**) Porcine alveolar macrophages (PAMs); (**B**) Lung; (**C**) Spleen; (**D**) Inguinal lymph nodes. “LW” represents Large White pigs and “TC” represents Tongcheng pigs. Student’s *t*-test: * *p* < 0.05.

In this study, we performed quantitative reverse transcription PCR (qRT-PCR) to detect the expression change of porcine *ISG12A* mRNA in alveolar macrophages and immune related tissues, including spleen, lung and inguinal lymph nodes. The results showed that the expression of porcine *ISG12A* mRNA was up-regulated in cells and different tissues when pigs were challenged with PRRSV in vivo. Compared with negative controls, the relative mRNA expression of porcine *ISG12A* was up-regulated in PRRSV-challenged porcine alveolar macrophages (PAMs; fold change of 568.41 and *p*-value of 0.084 for Tongcheng (TC) pigs; fold change of 106.15 and *p*-value of 0.026 for Large White (LW) pigs), lung (fold change of 11.95 and *p*-value of 0.31 for TC pigs; fold change of 12.17 and *p*-value of 0.034 for LW pigs), spleen (fold change of 3.84 and *p*-value of 0.32 for TC pigs; fold change of 6.78 and *p*-value of 0.029 for LW pigs), inguinal lymph node (fold change of 13.25 and *p*-value of 0.010 for TC pigs; fold change of 12.05 and *p*-value of 0.13 for LW pigs) in both breeds (Figure 4A–D). Although statistical results were not significant for TC pigs, due to individual differences, we still found that TC pigs had same expression trend as LW pigs after PRRSV challenge.

### 2.4. Porcine ISG12A Restricted PRRSV Replication in MARC-145 Cells

**Figure 5:**
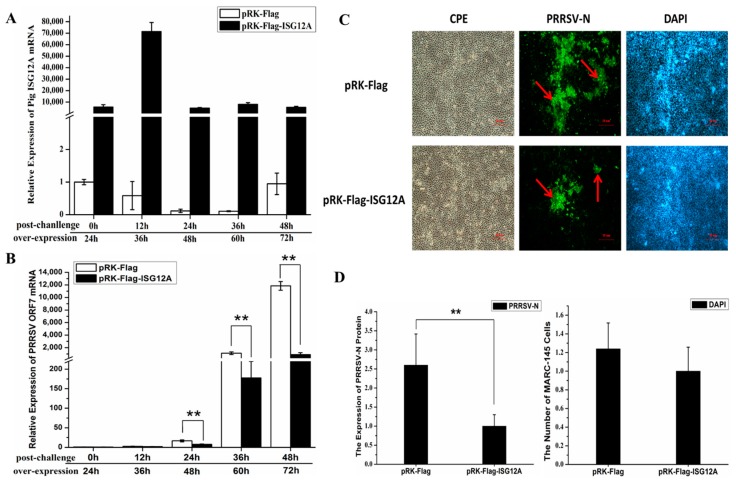
Ectopic expression of porcine ISG12A restricted PRRSV replication. The control vector (pRK-Flag) or pRK-Flag-ISG12A were transfected into MARC-145 cells, and PRRSV replication was assessed by expression of PRRSV *ORF7* mRNA and PRRSV N protein. (**A**) The relative mRNA level of porcine *ISG12A* at 0, 12, 24, 36 and 48 hpc; (**B**) The relative mRNA level of PRRSV *ORF7* at 0, 12, 24, 36 and 48 hpc; (**C**) Result of immunofluorescence assay. “CPE” indicates the observed cytopathic effects in the bright field, “PRRSV-N” represents the results of fluorescence-stained cells with the antibody against the PRRSV N protein (green marked by red arrows); “DAPI” represents the cell density by nuclear staining with DAPI (blue); scar bar = 10 µm; (**D**) The statistical results of the IFA and the number of fluorescence-positive cells were normalized to the total cell number (DAPI-stained cells) to evaluate the relative PRRSV level. Student’s *t*-test: ** *p* < 0.01.

To further investigate its antiviral ability, porcine ISG12A was ectopically overexpressed in MARC-145 cells for 24 h and then challenged with PRRSV at multiplicity of infection (MOI) of 0.1. After that, the cells were harvested at 0, 12, 24, 36 and 48 h post-challenge (hpc), respectively. The qRT-PCR results showed that the relative expression of porcine *ISG12A* mRNA was much higher compared with the control (Figure 5A), and PRRSV *ORF7* mRNA in ISG12A overexpressed MARC-145 cells was significantly lower than that in the control group at 24, 36 and 48 hpc (Figure 5B). In order to confirm the restriction effect of porcine ISG12A, immunofluorescence assay (IFA) was used to detect the expression level of PRRSV N protein. The results showed that the expression level of PRRSV N protein in ISG12A overexpressed MARC-145 cells was significantly lower than that in control group at 36 hpc which decreased approximately 2.6 times (*p*-value = 0.000235) (Figure 5C,D). Both results demonstrated overexpression of porcine ISG12A restricted PRRSV replication in MARC-145 cells. 

### 2.5. Knockdown of Endogenous ISG12A Enhanced PRRSV Replication in MARC-145 Cells 

**Figure 6:**
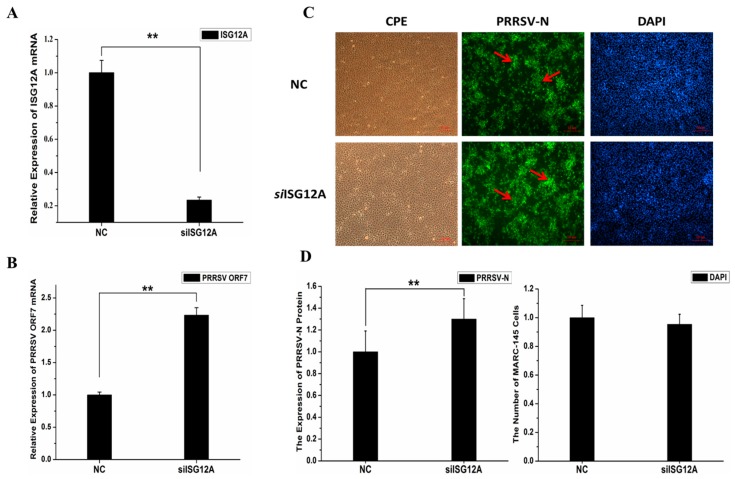
Decreased basal level of porcine ISG12A using siRNA promoted PRRSV replication. Nagtive control (NC) and siRNA were transfected into MARC-145 cells 24 h, after that the cells were infected with PRRSV, and the cells were collected at 36 hpc. (**A**) The relative level of porcine *ISG12A* mRNA in MARC-145 cells after 36 hpc; (**B**) The relative level of PRRSV *ORF7* mRNA in MARC-145 cells at 36 hpc; (**C**) Result of the IFA. “CPE” indicates the observed cytopathic effects in the bright field; “PRRSV-N” represents the results of fluorescence-stained cells with the antibody against PRRSV N protein (green marked by red arrows); “DAPI” represents the cell density by nuclear staining using DAPI (blue); (scar bar: 10 µm); (**D**) The statistical results of the IFA, the number of fluorescence positive cells were normalized to the total cell number (DAPI-staining cells) to evaluate the relative PRRSV level. Student’s *t*-test: ** *p* < 0.01.

To further prove the antiviral effect of porcine ISG12A, MARC-145 cells were transiently transfected with siRNA for 24 h, and then challenged by PRRSV (MOI of 0.1) for another 36 h. The qRT-PCR results showed that the expression of *ISG12A* mRNA significantly decreased (by approximately 77%, *p*-value = 6.61373 × 10^–5^) compared with that the control group (Figure 6A). Compared with the control group, the relative expression of PRRSV *ORF7* mRNA was significantly higher (fold change = 2.15; *p*-value = 0.00046) in siRNA-transfected MARC-145 cells (Figure 6B). The IFA results also confirmed that the level of PRRSV N protein in siRNA transfected MARC-145 cells were significantly higher than that in the control group, up-regulated approximately 1.3 times (*p*-value = 1.18564 × 10^−5^) (Figure 6C,D). Both results demonstrated that ISG12A could significantly influence PRRSV replication in MARC-145 cells.

## 3. Discussion

Innate immunity is the first line for pigs to defend against PRRSV infection. IFNs and ISGs are important components of innate immunity involved in inhibition of PRRSV replication [13,14,15,25]. Gjermandsen and colleagues (2000) reported that ISG12A could be induced by both IFN-I and IFN-γ [19]. Although PRRSV significantly inhibited expression of IFN-I and blocked the IFN-I signaling pathway, IFN-γ could be induced during the early stage of PRRSV infection [26]. We found that porcine *ISG12A* was the highest up-regulated gene in PAMs of both TC and LW pigs after PRRSV challenge (data unpublished). Recent studies have demonstrated that ISG12A could significantly suppress proliferation of HCV, NDV and VSV [21,22,23,27]. To the best of our knowledge, our present study is the first to confirm porcine *ISG12A* as a novel anti-PRRSV gene. Researches have proved that virus-mediated cell cycle regulation may enhance virus infection or pathogenesis [28,29]. Nonstructural Protein 11 (NSP-11) of PRRSV was reported to be critical for viral replication, significantly increasing the S phase cell percentage and slightly increased the G2 phase percentage in MARC-145 cells [30]. *Actinobacillus pleuropneumoniae* significantly inhibited PRRSV replication through arresting St. Jude porcine lung (SJPL) cells in G2/M phase [31]. To restrict PRRSV replication, many host genes related to cell cycle and DNA replication were up-regulated in highly pathogenic PRRSV (HP-PRRSV) infected PAMs [32]. In this study, porcine ISG12A was confirmed to have arrested cells in G2/M phase; this might be a strategy for host immune system to fight against PRRSV through changing the cell state to restrict PRRSV replication. On the other hand, ISG12A can induce cytochrome c releasing, Bax activation and mitochondrial membrane destabilization to enhance apoptosis [33,34]. In this study, we found that porcine ISG12A was located in mitochondria, which may contribute to promote apoptosis during PRRSV infection. Although PRRSV infection can delay host cell apoptosis at early stage of infection to facilitate proliferation and spread of PRRSV [35], porcine ISG12A may induce apoptosis by eliminating itself to prevent PRRSV replication and dissemination.

In conclusion, this study first presented anti-PRRSV ability of porcine ISG12A. Overexpression of porcine ISG12A inhibited PRRSV replication and knockdown of ISG12A promoted PRRSV replication. These results provide important information investigate host immune system responses to PRRSV infection, and lay a strong foundation for new therapy development to control PRRSV.

## 4. Materials and Methods

### 4.1. Cells, Tissues, and Virus

MARC-145 cells (green monkey fibroblast cell line) and PK-15 cells (porcine kidney cell line) were purchased from the China Center for Type Culture Collection (CCTCC) and cultured in Dulbecco’s Modified Eagle Medium (DMEM; Hyclone, South Logan, UT, USA) supplemented with 10% heat-inactivated fetal bovine serum (FBS) (Gibco™, Thermo Fisher Scientific, New York, NY, USA). PRRSV strain WUH3 (Accession: HM853673.2, NCBI), a highly pathogenic PRRSV strain, was kindly provided by Shaobo Xiao (Huazhong Agricultural University, Wuhan, China) [36]. 

Six Tongcheng pigs and six Large White pigs 5 weeks of age verified to be negative for PRRSV, pseudorabies virus (PRV) and porcine circovirus type 2 (PCV2) were randomly and equally assigned into two groups, respectively. The challenge group was challenged with PRRSV WUH3 (3 mL/15 kg; 10^−5^ TCID_50_/mL) by intramuscular inoculation, and the control group was challenged with DMEM. All piglets were slaughtered on the seventh day post-challenge. PAMs and pig tissues used in this study from piglets were stored at −80 °C [37].

### 4.2. Sequence Analysis

The conserved domain was analyzed by online NCBI Conserved Domains Database (CDD) (https://www.ncbi.nlm.nih.gov/Structure/cdd/wrpsb.cgi, CDD version 3.16-50369 PSSMs). Clustw2.1 online software (http://www.ebi.ac.uk/Tools/msa/clustalw2) was used to perform multiple sequence alignment of porcine ISG12A and the other eight species homologues. The phylogenetic tree was constructed with the minimum-evolution method by MEGE6.0 [38].

### 4.3. Expression Vectors

The full-length coding sequence (CDS) of porcine ISG12A was amplified from PAMs of PRRSV infected TC pigs and was performed in 50 µL amplification reaction containing 2 µL of cDNA, 2 µL forward and reverse primers (10 pmol), 10 µL 5× PrimeSTAR GXLBuffer, 4 µL 2.5 nM dNTPs, 1 µL PrimeSTAR^®^GXL DNA Polymerase (TAKARA, Tokyo, Japan) and 29 µL ddH_2_O. The reaction procedure was performed at 98 °C for 3 min, with a following 36 cycles at 98 °C for 5 s, 60 °C for 30 s, 72 °C for 1 min, and finally 72 °C for 5 min. The CDS of porcine ISG12A was then cloned into pEGFP-N1 or pRK-Flag vectors. The primer pairs used for PCR reaction were listed in Table 1.

### 4.4. Confocal Microscopy

MARC-145 or PK-15 cells were seeded onto coverslips in 12-well plate (Costar, New York, NY, USA) for 12 h and transfected with 1.6 µg pEGFP-N1-ISG12A or control using Lipofectamine 2000 (Invitrogen, Carlsbad, CA, USA) according to the manufacturer’s instruction. After 24 h post-transfection, the cells were washed with PBS three times. The mitochondria were then stained with MitoTracker^®^Red CM-H2XRos (Invitrogen, Carlsbad, CA, USA) for 20 min at 37 °C. The washed cells were fixed with 4% paraformaldehyde in PBS for 15 min after washing with PBS three times at room temperature. The nuclei were stained with 1 µg/mL 4′,6-diamidino-2-phenylindole (DAPI; Beyotime, Shanghai, China) for 3 min. The coverslips were mounted on microscope slides with fluorescence mounting medium and subjected to confocal microscopy. Images were obtained under the eyepiece (10×) × objective (40×) magnification by adjusting the captured scale using an FV1000 (OLYMPUS, Tokyo, Japan) laser scanning confocal microscope.

### 4.5. Flow Cytometry

MARC-145 cells were seeded onto coverslips in 6-well plates (Costar, New York, NY, USA) for 16 h and transfected with 1.6 µg pEGFP-N1-ISG12A or the control using Lipofectamine 2000 (Invitrogen, Carlsbad, CA, USA) according to the manufacturer’s instructions. The cells were then trypsinized, washed with PBS, and suspended in cold PBS at a density of 1 × 10^6^ cells/mL. The cells were harvested by centrifugation at 1000× *g* for 5 min, and then fixed with 70% cold ethanol at 4 °C for 2 h. The cells were washed with cold PBS twice, and stained with 10 µg/mL propidium iodide (PI) (Beyotime, Shanghai, China) prepared in PBS containing 0.1% Triton X-100 and 10 µg/mL RNaseA for 30 min at room temperature in the dark. The samples were analyzed by flow cytometry (FACS Calibur, BD, Franklin Lakes, NJ, USA) and the data were analyzed with Modfit LT software (ModFit LT 2.0, Verity Software House Company, Augusta, ME, USA). 

### 4.6. Gene Expression in Cells and Tissues during PRRSV Challenge In Vivo

As presented in previous paper [37], TC pigs and LW pigs were challenged with the PRRSV WHU3 strain, and slaughtered on 7 days post-challenge. The uninfected pigs were treated as the control group. The total RNA was extracted from the PAMs, spleens, lungs and lymph nodes using TRIZOL (Invitrogen, Carlsbad, CA, USA) according to the manufacturer’s instructions. One microgram of total RNA was used to synthesize cDNA with the PrimeScript RT reagent kit with the gDNA Eraser (TAKARA, Tokyo, Japan) according to the manufacturer’s instructions. The relative expression of genes was detected using qRT-PCR, using the SYBR Green Real-time PCR Master Mix (TOYOBO, Tokyo, Japan). The gene expression level was normalized according to the expression of GAPDH. The 2^−ΔΔ*C*t^ method was used to quantify the relative changes in gene expressions [39]. The primer pairs used for the qRT-PCR were listed in Table 1.

### 4.7. Transient Transfection and Real-Time Quantitative PCR

MARC-145 cells were seeded into 12-well plates (Costar, New York, NY, USA). When the cells reached 70–80% confluence, four micrograms empty or recombinant vectors were transfected separately into the MARC-145 cells using Lipofectamine2000 (Invitrogen, Carlsbad, CA, USA) according to the manufacturer’s manual. Twenty-four hours later, the MARC-145 cells were challenged with PRRSV (MOI = 0.1) and incubated for 1 h. After removal of the excess virus, the MARC-145 cells were incubated in cell culture medium containing 3% FBS for 0, 12, 24, 36 and 48 h, respectively, and then harvested. 

Three endogenic *ISG12A* siRNAs and the control siRNA were designed and synthesized by Shanghai GenePharma company (Shanghai, China). MARC-145 cells were seeded into 12-well plates (Costar, New York, NY, USA). When the cells reached 70–80% confluence, ISG12A (or NC) siRNA were transfected respectively into the MARC-145 cells using Lipofectamine 2000 (Invitrogen, Carlsbad, CA, USA) according to the manufacturer’s manual. Twenty-four hours later, the MARC-145 cells were challenged with PRRSV (MOI = 0.1) and incubated for 1 h. After removal of the excess virus, the MARC-145 cells were incubated in cell culture medium containing 3% FBS for 36h, and then harvested.

Total RNA extraction, cDNA synthesis and qRT-PCR analysis were performed as was described in Section 4.6. The primer pairs used for qRT-PCR were listed in Table 1.

### 4.8. Immunofluorescence Assay

MARC-145 cells were seeded into 6-well cell plates (Costar, New York, NY, USA). When cells reached nearly 70–80% confluence, four micrograms recombinant or control vectors were transfected respectively into the MARC-145 cells using Lipofectamine2000 (Invitrogen, Carlsbad, CA, USA) according to the manufacturer’s instructions. Twenty-four hours later, the cells were challenged with PRRSV (MOI = 0.1) and incubated for 1 h, which was followed by removal of the excess virus and 36 h of incubation in cell culture medium containing 3% FBS. Subsequently, the MARC-145 cells were washed three times with PBS and fixed in 4% paraformaldehyde solution for 15 min, followed by incubation with blocking buffer (PBS containing 5% bovine serum albumin (BSA) and 0.5% TritonX-100) for 1 h, and anti-PRRSV N protein monoclonal antibodies SDOW-17A (1:1000) (Brookings, SD, USA) at 4 °C overnight. Next, the MARC-145 cells were washed with PBS, and incubated with Alexa Fluor 488-labeled goat anti-mouse immunoglobulin G (IgG) (Beyotime, Shanghai, China) at room temperature for 1 h. Nuclei were stained with DAPI (Beyotime, Shanghai, China) for 3 min. After washing with PBS three times, cells were observed on fluorescent microscope (TE2000-S, Nikon, Tokyo, Japan). Twenty random visual fields per treatment group were captured to calculate the number of MARC-145 cells based on DAPI signal and the PRRSV-N protein expression level according to green fluorescence by Image-pro plus 6.0 (Ipp6.0) software (Media Cybernetics, Silver Springs, MD, USA).

### 4.9. Statistical Tests

The results were presented as mean ± standard deviation (SD). Statistical significance was assessed by Student’s *t*-test, with *p* < 0.05 considered as statistically significant and *p* < 0.01 statistically highly significant.

## Figures and Tables

**Table 1:** Oligos used in the present study.

Gene	Primer Sequence (5′–3′)	*T* (°C)	Application
p*ISG12A*-PF1	ATCTCGAGTATGGCCATGGTAGCCACCC	58	Expression vector construction
p*ISG12A*-PR1	GTAAGCTTCAAGCCCACTATTTTGGTAC		
p*ISG12A*-PF2	GCCACCCAAGCAGTCTTATCCTC	60	qRT-PCR
p*ISG12A*-PR2	TGTCCCCAGCAAGGATCCCA		
m*ISG12A*-PF3	GTGGCCTCCGCTTTCACCT	60	qRT-PCR
m*ISG12A*-PR3	TTCCCGTCGCAGTGAAACCC		
*ORF7*-PF	CCCATTTCCCTCTAGCGACT	60	qRT-PCR
*ORF7-PR*	GCGTCGGCAAACTAAACTCCA		
m*GAPDH*-PF	TGGGGAAGGTGAAGGTCGG	60	qRT-PCR
m*GAPDH*-PR	TCCTGGAAGATGGTGATGGG		
p*GAPDH*-PF	CGTCCCTGAGACACGATGGT	60	qRT-PCR
p*GAPDH*-PR	GCCTTGACTGTGCCGTGGAAT		
*ISG12A*-siRNA	GCUUUCACCUCAUCAGCAGUG		Macaque *ISG12A* siRNA
NC-siRNA	UUCUUCGAACGUGUCACGUTT		Negative control siRNA

p: pig; m: macaque.
